# A Double-Yield-Surface Elastoplastic Constitutive Model for Cemented Sand and Gravel Dam Materials

**DOI:** 10.3390/ma19204397

**Published:** 2026-10-19

**Authors:** Fan Li, Minmin Jiang, Jie Yang

**Affiliations:** 1 China Three Gorges Corporation, Wuhan 430014, China; li_fan6@ctg.com.cn; 2 School of Transportation and Civil Engineering, Nantong University, Nantong 226019, China; yangjie110@ntu.edu.cn

**Keywords:** cemented sand and gravel, CSG dam, double-yield surface, generalized plasticity, constitutive model

## Abstract

Cemented sand and gravel (CSG) exhibits distinct shear and volumetric deformation responses. This study assesses a double-yield-surface incremental formulation and recalibrates its axial and volumetric response relations using coordinates digitized from the available triaxial plots. Three binder contents, 20, 60, and 100 kg/m^3^, are examined at confining pressures of 300–1200 kPa. Stress-induced yield differentials, plastic multipliers, and the coefficients used for triaxial path matching are distinguished. Mixture-specific parameters and yield-contour shape ratios are reported, and the necessary axial condition *E*_t_ ≤ *E* is satisfied over the investigated ascending branches. The pointwise calibration root-mean-square residuals range from 31 to 131 kPa in deviatoric stress and from 0.021 to 0.179 percentage points in volumetric strain. These are in-sample fitting statistics, not independent prediction errors or estimates of specimen-to-specimen variability. Published test methods are briefly summarized, while specimen-level records and replicate variability associated with the digitized curves remain uncertain. The volumetric-hardening relation requires additional source-state information for independent reconstruction. Accordingly, the findings support an assessment of graphical calibration within the reported stress and strain ranges, rather than independent validation or completed numerical model implementation.

## 1. Introduction

Cemented sand and gravel (CSG) is produced by mixing locally available sand and gravel, crushed rock, or suitable excavated material with a limited amount of cementitious binder and water. It combines features of a granular skeleton and cemented contacts. CSG and related hardfill dam concepts seek to match structural form to local materials and construction conditions [1,2,3,4]. Physical modeling and multiscale studies demonstrate the importance of representing material deformation in CSG dam analyses [5]. Reliable constitutive characterization is therefore relevant to engineering design, although material-scale agreement alone does not establish structural-scale predictive performance.

Early stress–strain descriptions used modified Duncan–Chang, generalized hyperbolic, and other nonlinear relationships [6,7,8,9]. These models provide convenient response fits, but a reversible nonlinear elastic relation cannot, by itself, represent accumulated irreversible strain. Elastoplastic descriptions of cemented clays, CSG, cemented coarse-grained materials, and cement-mixed gravelly soils address yielding and cementation more explicitly [10,11,12,13]. The role of bond degradation also motivates damage hypoplasticity and statistical damage formulations [14,15]. Reviews emphasize the diversity of mixtures and the resulting difficulty of transferring one parameter set between projects [16].

Recent international work broadens this context. Luo et al. [17] developed a cemented granular model with bond degradation, and Rossi et al. [18] considered lightly cemented granular materials with a micromechanical description. Chen et al. [19] proposed a unified generalized plasticity description of cemented and uncemented sand–gravel materials. Ma et al. [20] incorporated bonding and filling into an elastoplastic CSG model. These developments complement the strength-based CSG formulation of Wu et al. [21], which also uses shear and volumetric surfaces and addresses softening and dilation. Thus, neither the use of two surfaces nor the distinction between shear and volumetric mechanisms is unique to the present work.

The present work focuses on the calibration and analytical consistency of an incremental double-yield-surface formulation for CSG. It combines the shear-surface construction of Cai et al. [11] with mixture-specific stiffness, strength, characteristic axial-strain, and volumetric-transition relations. The same response-calibration framework is examined at binder contents of 20, 60, and 100 kg/m^3^. The axial and volumetric response functions enter an explicit two-surface coefficient reduction for conventional drained triaxial loading, while the derivation identifies the stress-derivative conventions, admissibility conditions, and additional information needed to define volumetric hardening. The assessment is restricted to the available pre-peak graphical records; it does not establish independent validation or a new damage mechanism.

## 2. Relation to Existing Constitutive Models

Classical work-hardening plasticity [22], generalized plastic potential theory [23], and generalized plasticity [24,25,26] provide different routes to incremental descriptions of geomaterials. These frameworks should not be conflated. Here, explicit yield functions and plastic potentials are retained, and the two loading coefficients are selected from prescribed triaxial tangent relations. This is a path-based construction within the generalized plastic potential framework; it is not a numerical verification of a fully integrated three-dimensional generalized plasticity model.

Critical-state formulations [27,28], the Lade–Duncan model [29], and models discussed by Yin, Li, and Shen [30,31,32] illustrate different treatments of pressure dependence and dilatancy. Their capabilities depend on their particular yield, flow, and hardening laws. No general claim is made that all Cam-Clay-type models are unable to dilate or that all established soil models necessarily fail for CSG. Transfer to a cemented aggregate mixture requires calibration and examination of the relevant mechanisms.

Table 1 distinguishes the principal CSG comparisons. Parameter counts are quoted only where a comparable count is established; counts for damage and hypoplastic models depend on which elastic, damage, and state quantities are included. No ranking of complexity or accuracy is inferred from an unmatched parameter count. A direct performance comparison would require a common dataset, common strain range, and equivalent calibration information.

## 3. Double-Yield-Surface Incremental Formulation

### 3.1. Stress, Strain, and Modeling Assumptions

Compression is positive. Under axisymmetric conventional triaxial compression, *σ*_1_ is the axial effective principal stress and *σ*_2_ = *σ*_3_ is the radial effective principal stress. The stress invariants are *p* = (*σ*_1_ + 2*σ*_3_)/3 and *q* = *σ*_1_ − *σ*_3_. Axial and radial strains are *ε*_a_ = *ε*_1_ and *ε*_r_ = *ε*_2_ = *ε*_3_. The volumetric strain is *ε*_v_ = *ε*_a_ + 2*ε*_r_, and the work-conjugate shear strain is *ε*_s_ = 2(*ε*_a_ − *ε*_r_)/3 = *ε*_a_ − *ε*_v_/3. Consequently, *σ*:d*ε* = *p* d*ε*_v_ + *q d*ε_s_ in axisymmetric loading. Positive *ε*_v_ denotes contraction and negative increments denote dilation.

The formulation assumes small strain, an isotropic elastic response, drained monotonic compression, and separately identifiable shear and volumetric loading mechanisms. It does not contain a bond-damage variable, rate dependence, aging, or a calibrated post-peak law. Associated plastic potentials are used as a simplifying assumption. The intended branch is the ascending response with a positive tangent modulus; the exact peak, where the prescribed tangent vanishes, requires separate numerical treatment. Binder content enters through separately calibrated parameter sets, rather than a verified continuous evolution law.

Strains used in the axial and volumetric shear-response fits are measured relative to the end of isotropic consolidation. Plastic state variables accumulated during consolidation are retained separately. Resetting the plotted shear-strain origin must not reset the volumetric-hardening state.

### 3.2. Elastic Response

The isotropic elastic law is written in tensor form to avoid ambiguity over tensorial and engineering shear strains:

(1) $$\mathrm{d\sigma}=K\;\mathrm{tr}({\mathrm{d\varepsilon}}^{e})\;I\;+\;2G_{e}\;\mathrm{dev}({\mathrm{d\varepsilon}}^{e}),\;\;G_{e}\;=\;\frac{E}{2(1+\nu )}$$

Here, *K* is the elastic bulk modulus, *G*_e_ is the elastic shear modulus, *I* is the identity tensor, and *E* is the unloading–reloading Young modulus. In a Voigt form with engineering shear strains γ_ij_ = 2ε_ij_, the shear diagonal entries are *G*_e_; using tensorial shear-strain components gives 2*G*_e_. Equation (1) is the standard isotropic elastic identity, not a new constitutive relationship.

(2) $$E=E_{e}=K_{\mathrm{ur}}E_{0}P_{a}{\left(\frac{P_{a}+\sigma_{3}}{P_{a}}\right)}^{n}$$

(3) $$\nu =\frac{1}{2}-\frac{E}{6K}$$

(4) $$K=\frac{\mathrm{dp}}{{{\mathrm{d\varepsilon}}_{v}}^{e}}=\frac{p_{0}+P_{a}}{k}$$

Equation (2) is an empirical pressure-dependent modulus relationship used in the CSG calibration framework [11], with related modulus fitting in [6,7,8,9]. Equation (3) follows from the standard isotropic elasticity identity *E* = 3*K*(1 − 2*ν*); the corresponding algebra is summarized in Appendix A. Equation (4) uses the logarithmic unloading–reloading interpretation adopted in [11,33]. *P*_a_ = 100 kPa is a fixed reference pressure. *E*_0_ is dimensionless because it multiplies *P*_a_; the initial modulus at zero confining pressure is *E*_0_*P*_a_. The exponent *n* and the unloading ratio *K*_ur_ are dimensionless. The latter relates *E* to the initial loading modulus *E*_i_ in Equation (27). The reported *K*_ur_ and *k* values are retained; the response parameters are recalibrated as described in Section 3.8. This recalibration changes the inferred *E* and does not constitute a new unloading experiment.

In Equation (4), *p*_0_ is the reference consolidation pressure for the shear stage. Along that stage, the stated calibration uses *K* evaluated at *p*_0_. During isotropic unloading–reloading, the corresponding local form is *K*(*p*) = (*p* + *P*_a_)/*k*, obtained from d*ε*_v_^e^ = *k* dln[(*p* + *P*_a_)/*P*_a_]. The parameter *k* is a dimensionless logarithmic elastic-compression slope, rather than a modulus. Tabulated percentage values must be converted to decimal strain before use.

An admissible elastic state requires *E* > 0, *K* > 0, and −1 < *ν* < 0.5, equivalently 0 < *E* < 9*K*. A slightly negative *ν* is not, by itself, a violation of isotropic elastic positive definiteness, although it requires experimental support for the material in question. No elastic constant is silently clipped to a chosen range.

### 3.3. Yield Functions and Their Provenance

Yield-surface construction may use stress–dilatancy relations, critical-state ideas, equal-plastic-strain contours, or empirical fitting [27,28,29,30,31,32,34,35]. The following shear construction is inherited from [11]; the pressure shift and response fits are empirical adaptations. Standard tensor identities and the derivative reductions below are derived explicitly. These different origins are kept distinct.

#### 3.3.1. Shear Surface

For compactness and a finite *q* → 0 evaluation, the inherited shear function is written in a rationalized form:

(5) $$f_{1}\;=\;\frac{2{\tilde{\varepsilon }}_{\mathrm{sm}}B_{s}}{A_{s}+2B_{s}+\sqrt{{A_{s}}^{2}+4A_{s}B_{s}}}\;-\;\frac{q}{3K_{\mathrm{ur}}G_{i}}\;-\;\kappa_{s}\;=\;0$$

(6) $$A_{s}=1-\frac{q}{q_{f}},\;B_{s}=\frac{q}{3G_{i}{\tilde{\varepsilon }}_{\mathrm{sm}}}$$

The rationalization is algebraically equivalent to the square-root form in [11] and avoids subtraction of nearly equal terms near *q* = 0. Its continuous value at *q* = 0 is *f*_1_ = −κ_s_. The scalar κ_s_ is the shear hardening coordinate inferred by elastic subtraction in the inherited construction. It is distinguished here from the plastic multiplier and from the work-conjugate plastic strain calculated by the flow rule.

The term *q*/(3*K*_ur_*G*_i_) is an empirical elastic-subtraction approximation in that construction. It equals the physical elastic shear strain *q*/(3*G*_e_) only if *K*_ur_*G*_i_ = *G*_e_ for the applicable state and modulus convention. Because *G*_i_ is an empirical response scale, that equality cannot be assumed from Equation (3). Identifying κ_s_ exactly with *ε*_s_^p^ therefore needs an additional compatibility check. This limitation is relevant to transferring the calibrated surface to other stress paths.

(7) $$q_{f}=M(p+p_{r}),\;M=\frac{6\;\mathrm{sin \varphi}}{3-\mathrm{sin \varphi}},\;\;p_{r}=c\;\mathrm{cot \varphi}$$

Equation (7) is the triaxial-compression form of the Mohr–Coulomb strength relation. The parameter *p*_r_ is a positive pressure offset when *c* > 0; the actual intercept of the straight line on the *p*-axis is −*p*_r_. The envelope value *q*_f_ depends on the current *p* and is not generally a constant along a conventional triaxial path. At failure on a path with fixed *σ*_3_, the corresponding target stress is *q*_m_ = 2(*c* cos φ + *σ*_3_ sin φ)/(1 − sin φ), derived explicitly in Appendix A. A fitted target must not be confused with a measured peak where the experimental curve has not reached failure.

(8) $$G_{i}=G_{0}P_{a}{\left(\frac{p+P_{a}}{P_{a}}\right)}^{n_{g}},\;\;G_{0}=\frac{E_{0}}{2},\;\;n_{g}=n$$

Equation (8) is an empirical pressure-dependent shear-response scale following [11,30]. *G*_0_ is dimensionless, and *G*_0_*P*_a_ is its zero-pressure value. The modulus-factor and exponent mapping in Equation (8) is a calibration simplification; *G*_i_ is not the elastic modulus *G*_e_ in Equation (1). Partial derivatives of Equation (5) must retain the dependence of both *q*f and *G*_i_ on *p*.

(9) $${\tilde{\varepsilon }}_{\mathrm{sm}}=\frac{2}{3}\varepsilon_{m},\;\;\varepsilon_{\mathrm{sm}}=\varepsilon_{m}-\frac{\varepsilon_{v}(\varepsilon_{m})}{3}$$

The first quantity in Equation (9) is the auxiliary peak-strain scale used in Equation (5), not an exact invariant transformation. The second is the work-conjugate shear strain at the axial target. The two coincide only when *ε*_v_(*ε*_m_) = *ε*_m_. For a given calibrated path, the auxiliary scale is held fixed when taking stress gradients; a mixture-level average may also be used only if that convention is stated and tested. A general stress-path formulation requires a consistent state-dependent calibration of this scale.

(10) $$\varepsilon_{m}=\lambda_{0}\frac{\sigma_{3}}{P_{a}}+d_{0}$$

Equation (10) is an empirical regression for the characteristic axial strain. *λ*_0_ is its slope with respect to normalized confining pressure, and *d*_0_ is the extrapolated zero-confinement intercept. The value at *σ*_3_ = *P*_a_ is *λ*_0_ + *d*_0_. For a continuously hardening low-binder curve, *ε*_m_ is a fitted characteristic target unless a peak has actually been observed.

#### 3.3.2. Volumetric Surface and Initial State

The volumetric surface uses a cementation pressure shift in a Cam-Clay-type construction [11,27,28]:

(11) $$f_{2}\;=\;p\;+\;\frac{q^{2}}{{M_{1}}^{2}(p+p_{r})}\;-\;p_{c}\;=\;0$$

The domain is *p* + *p*_r_ > 0. The hardening pressure *pc* depends on accumulated plastic volumetric strain, including consolidation. A monotone relation *H*_v_ is introduced in the logarithmic pressure coordinate *ξ*:

(12) $$\xi \;=\;\ln(1+\frac{p_{c}}{P_{a}}),\;{\varepsilon_{v}}^{p}\;=\;H_{v}(\xi ),\;p_{c}\;=\;P_{a}[\exp({H_{v}}^{-1}({\varepsilon_{v}}^{p}))-1]$$

For an interval between successive hardening knots, the interpolation is

(13) $$H_{v}(\xi )={\varepsilon_{i}}^{p}+h_{i}(\xi -\xi_{i}),\;h_{i}=\frac{{\varepsilon_{i+1}}^{p}-{\varepsilon_{i}}^{p}}{\xi_{i+1}-\xi_{i}}>0$$

**Figure 1:**
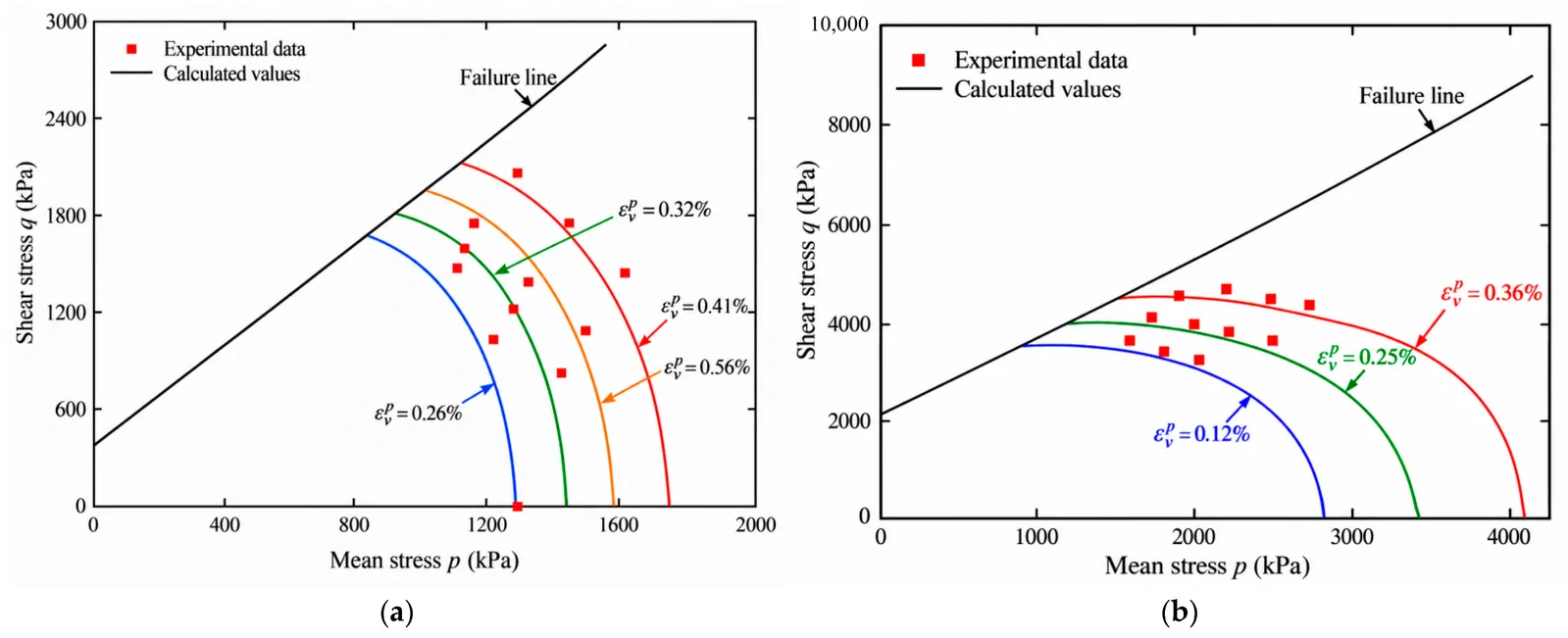
Graphical calibration of volumetric yield contours for CSG with binder contents of (**a**) 20 kg/m^3^ and (**b**) 100 kg/m^3^. Symbols denote digitized points from the available source plots, and curves denote fitted yield loci. These comparisons do not constitute independent validation.

This piecewise relation generalizes the constant plastic logarithmic slope h_v_ = *k*′ − k_e_ used in the inherited description. A constant h_v_ is recovered when all segment slopes *h*_i_ are equal; these slopes cannot be inferred from the elastic unloading parameter *k* alone. The equal-plastic-volumetric-strain labels and yield-locus markers displayed in Figure 1 provide graphical constraints on the contours, but the numerical pressure–strain knot list and its laboratory provenance are not preserved in the present submission. Consequently, Equation (13) specifies the interpolation procedure, rather than a fully reproducible material-specific hardening dataset. The contours must not be interpreted as independently measured isotropic hardening curves; quantitative state-update calculations require independently documented knots and initial hardening states.

The shape factors are fitted separately for the two available contour datasets. The estimated *M*_1_/*M* values are 1.3469 for 20 kg/m^3^ and 1.1704 for 100 kg/m^3^. The fixed value 1.3 is therefore replaced by mixture-specific contour estimates. The arithmetic midpoint, 1.2587, is used only as an assumed value for the unmeasured 60 kg/m^3^ contour case. The variation demonstrates why a universal shape ratio cannot be asserted from these plots. Neither a confidence interval nor experimental scatter is inferred from the fitted ratios.

To avoid erroneous joins and polygonal endpoints, the yield contours are plotted directly using the exact ellipse parameterization:

(14) $$p\;=\;\frac{p_{c}-p_{r}}{2}+\frac{p_{c}+p_{r}}{2}\;\mathrm{cos \theta},\;q\;=\;\frac{M_{1}(p_{c}+p_{r})}{2}\;\mathrm{sin \theta}$$

Only the compressive branch below the fitted strength envelope is displayed. Its intersection with *q* = *M*(*p* + *p*_r_) is *p* = (*M*_1_^2^*p*_c_ − *M*^2^*p*_r_)/(*M*_1_^2^ + *M*^2^). The curve reaches *q* = 0 continuously at *p* = *p*_c_; the vertical tangent there is a property of the stated equation. The same *c* and *φ* values are used in the response targets and in the envelope in Figure 1. Experimental marker coordinates are retained without smoothing or relocation.

For an initially isotropic consolidated state lying on the volumetric surface, *q* = 0, *p* = *p*_0_, and:

(15) $$p_{c0}\;=\;p_{0},\;{\varepsilon_{v0}}^{p}\;=\;H_{v}[\ln(1+\frac{p_{0}}{P_{a}})]$$

An initially overconsolidated state instead requires *p*_c0_ > *p*_0_, established from its loading history. Setting *ε*_v_^p^ = 0 in the zero-origin relation gives *pc* = 0; it does not establish a finite virgin yield pressure for a bonded specimen. A finite virgin yield pressure and the actual strain origin require independent state characterization. Resetting the plotted shear-strain origin must not erase plastic strain accumulated during consolidation.

Figure 1 illustrates graphical contour fitting for the 20 and 100 kg/m^3^ mixtures. The observed lower plastic volumetric-strain levels in the higher-binder panel may be consistent with reduced irreversible compression, but they do not establish purely elastic behavior or a general binder-content threshold. The contour markers and their fitted geometry provide only an in-sample assessment; they do not quantify specimen-level scatter. Because the pressure–strain hardening knots are not provided, the associated hardening law cannot be independently reconstructed from this figure alone.

### 3.4. Plastic Potentials and Increment Definitions

The associated choice *g*_1_ = *f*_1_ and *g*_2_ = *f*_2_ provides explicit flow directions without adding separately fitted plastic-potential parameters. It is a simplifying closure rather than a demonstrated physical law for CSG. For frictional cemented aggregates, tying yield geometry to flow direction can bias dilation. Multiple associated surfaces and fitted coefficients may reproduce a selected triaxial response, but this does not establish the correct volumetric response at a different stress path or confinement.

Let *n*_i_ = ∂*f*_i_/∂*σ* and *m*_i_ = ∂*g*_i_/∂*σ*, with the internal variables α held fixed. In a work-conjugate vector notation, the increment is

*dε* = *C^e^ dσ* + *m*_1_ *dΛ*_1_ + *m*_2_ *dΛ*_2_ (16)

(17) $$d_{\sigma }f_{i}={n_{i}}^{T}d\sigma ,\;\;d\Lambda_{i}=K_{i}d_{\sigma }f_{i}\;\;(\mathrm{active}\;\mathrm{surface})$$

(18) $$df_{i}=d_{\sigma }f_{i}+\frac{\partial f_{i}}{\partial \alpha }\cdot \mathrm{d\alpha}=0\;\;(\mathrm{consistency})$$

C^^e^^ is the elastic compliance. The plastic multiplier increment is d*Λ*_i_; *K*_i_ is a scalar path-matching coefficient and is not itself a multiplier. The symbol d_*σ*_f_i_ means only the stress-induced differential. It is generally nonzero during active loading, whereas the full differential d*f*_i_ is zero on a consistently evolving yield surface. Substituting the full d*f*_i_ for d_*σ*_f_i_ in Equation (17) would incorrectly eliminate active plastic flow. The units of *K*_i_ depend on the normalization of *f*_i_: *f*_1_ has strain units, whereas *f*_2_ has stress units. A single assumed unit for both coefficients is therefore inappropriate.

Equation (17) is the diagonal coefficient prescription used for the conventional triaxial reduction. A general two-surface state update additionally requires the coupled hardening consistency system discussed in Section 3.6. Non-negative *K*_i_ and outward loading are conditions to check, not consequences of a graphical fit.

### 3.5. Triaxial Coefficient Reduction and Response Functions

During conventional triaxial shear, d*σ*_2_ = d*σ*_3_ = 0, d*q* = d*σ*_1_, and d*p* = d*q*/3. Therefore, the derivative with respect to *σ*_1_ is a principal-stress derivative along this path. Applying the chain rule, with the calibrated auxiliary strain scales fixed, gives

(19) $$R_{1}=\frac{\partial f_{1}}{\partial \sigma_{1}}=\frac{1}{3}\frac{\partial f_{1}}{\partial p}+\frac{\partial f_{1}}{\partial q},\;\;R_{2}=\frac{1}{3}\frac{\partial f_{2}}{\partial p}+\frac{\partial f_{2}}{\partial q}$$

(20) $$R_{3}=\frac{\partial f_{1}}{\partial p},\;\;R_{4}=\frac{\partial f_{2}}{\partial p}$$

The partial derivatives of *f*_1_ and *f*_2_ with respect to *p* are taken at fixed *q* and fixed internal variables. In particular, for the volumetric surface of Equation (11), ∂*f*_2_/∂*p* = 1 − *q*^2^/[*M*_1_^2^(*p* + *p*_r_)^2^] and ∂*f*_2_/∂*q* = 2*q*/[*M*_1_^2^(*p* + *p*_r_)]. The corresponding derivatives of *f*_1_ retain the *p*-dependence of both *q*f and *G*_i_. Define *A* = 1/*E*_t_ − 1/*E* and *B* = μ_t_/*E*_t_ − 1/(3*K*). Axial and volumetric path matching then gives

(21) $$K_{1}{R_{1}}^{2}+K_{2}{R_{2}}^{2}=A,\;K_{1}R_{1}R_{3}+K_{2}R_{2}R_{4}=B$$

(22) $$K_{1}=\frac{AR_{4}-BR_{2}}{R_{1}(R_{1}R_{4}-R_{2}R_{3})},\;K_{2}=\frac{BR_{1}-AR_{3}}{R_{2}(R_{1}R_{4}-R_{2}R_{3})}$$

Here *E*_t_ = d*q*/d*ε*_a_ is the total loading tangent and μ_t_ = d*ε*_v_/d*ε*_a_ is the total volumetric-to-axial tangent ratio. Both differ from elastic moduli and from purely plastic dilatancy. The coefficient expressions in Equation (22) follow by solving the two linear equations in Equation (21), as summarized in Appendix A. The two-coefficient prescription applies when both surfaces are active; if only one is active, two arbitrarily specified tangents need not be attainable using that single flow direction.

The denominator is nonzero only if *Δ* = *R*_1_*R*_2_(*R*_1_*R*_4_ − *R*_2_*R*_3_) ≠ 0. A zero or poorly conditioned *Δ* invalidates this closed-form reduction. In addition, *K*_1_ ≥ 0 and *K*_2_ ≥ 0 must both be verified. A necessary condition on a positive-tangent branch is *A* ≥ 0, or 0 < *E*_t_ ≤ *E*. This condition is not sufficient: the volumetric target and the two flow directions must also be compatible. Negative coefficients must not be silently replaced by zero, because doing so changes the prescribed response.

#### 3.5.1. Axial Rational Interpolation and E_t_

The stress–strain interpolation used for path matching is an empirical rational response fit of the type used in nonlinear CSG characterization [6,7,8,9]:

(23) $$q(\varepsilon_{a})\;=\;\frac{\varepsilon_{a}}{a_{h}+b_{h}\varepsilon_{a}+c_{h}{\varepsilon_{a}}^{2}}$$

(24) $$E_{t}=\frac{\mathrm{dq}}{d\varepsilon_{a}}=\frac{a_{h}-c_{h}{\varepsilon_{a}}^{2}}{{\left(a_{h}+b_{h}\varepsilon_{a}+c_{h}{\varepsilon_{a}}^{2}\right)}^{2}}$$

(25) $$a_{h}=\frac{1}{E_{i}},\;b_{h}=\frac{1}{q_{m}}-\frac{2}{E_{i}\varepsilon_{m}},\;c_{h}=\frac{1}{E_{i}{\varepsilon_{m}}^{2}}$$

The subscripts h avoid confusion between the interpolation coefficient *c*_h_ and cohesion *c*. The three auxiliary coefficients are derived from *q*′(0) = *E*_i_, *q*(*ε*_m_) = *q*_m_, and *q*′(*ε*_m_) = 0; they are not additional independent material parameters. All three have inverse-stress units because strain is dimensionless. The algebraic derivation is provided in Appendix A.

For the scalar reconstruction in this paper, *q*_m_ is fixed at the Mohr–Coulomb target for each constant-*σ*_3_ test. This convention is essential. If *q*f(*p*) is updated continuously inside the stress–strain interpolation, Equation (23) is no longer its complete path derivative: the derivative of the moving strength target must also be included. Such an alternative requires a different derivation and a verified implementation.

(26) $$\varepsilon_{a}(q)=\frac{2a_{h}q}{1-b_{h}q+\sqrt{{\left(1-b_{h}q\right)}^{2}-4a_{h}c_{h}q^{2}}}$$

Equation (26) is the ascending inverse branch of Equation (24), written in a numerically stable form. It is defined for 0 ≤ *q* ≤ *q*_m_, with *ε*_a_(0) = 0. *E*_t_ > 0 for 0 ≤ *ε*_a_ < *ε*_m_ and *E*_t_ = 0 at *ε*_m_. Continuing the rational curve beyond *ε*_m_ would introduce a descending branch; that branch is excluded because post-peak softening has not been calibrated.

(27) $$E_{i}=E_{0}P_{a}{\left(\frac{P_{a}+\sigma_{3}}{P_{a}}\right)}^{n}$$

*E*_i_ is the initial loading tangent, not necessarily a purely elastic modulus. The unloading modulus is *E* = *K*_ur_*E*_i_. Distinguishing these quantities is necessary when evaluating the plastic coefficients.

#### 3.5.2. Volumetric Response and Tangent Ratio

*ε_v_* = *d*(*ε_a_* − *ε_n_*)^2^ + *ε_vd_* (28)

(29) $$\varepsilon_{n}=\lambda_{1}\frac{\sigma_{3}}{P_{a}}+d_{1}$$

(30) $$\varepsilon_{\mathrm{vd}}=\lambda_{2}\frac{\sigma_{3}}{P_{a}}+d_{2}$$

Equations (28)–(30) are empirical path fits. *ε*_n_ is the axial strain at maximum contraction, and *ε*_*vd*_ is that maximum contraction measured from the start of shearing. When *ε*_v_(0) = 0 and *ε*_n_ ≠ 0, *d* = −*ε*_*vd*_/*ε*_n_^2^, so *d* is an auxiliary coefficient rather than an additional independent parameter. A different strain origin would require an explicit offset. The *d*_1_ and *d*_2_ intercepts correspond to zero confining pressure; values at one atmosphere are *λ*_1_ + *d*_1_ and *λ*_2_ + *d*_2_, respectively.

The differentiation in Equation (31) is an ordinary derivative along the fixed-confining-pressure shear path. *σ*_3_, *ε*_n_, *ε*_*vd*_, *d*, and the mixture parameters are held constant. It is not a partial stress derivative or a tensor gradient:

(31) $$\mu_{t}\;=\;\frac{d\varepsilon_{v}}{d\varepsilon_{a}}\;=\;2d(\varepsilon_{a}-\varepsilon_{n})\;=\;-\frac{2\varepsilon_{\mathrm{vd}}}{{\varepsilon_{n}}^{2}}[\varepsilon_{a}(q)-\varepsilon_{n}]$$

Thus μ_t_ > 0 before maximum contraction, μ_t_ = 0 at *ε*_a_ = *ε*_n_, and μ_t_ < 0 afterward. The fitted transition strain changes with confinement; a universal transition stress ratio is not imposed. The total ratio μ_t_ differs from plastic dilatancy because elastic volume and axial increments must be subtracted. For the triaxial path, d*ε*_a_^p^/d*q* = A and d*ε*_v_^p^/d*q* = *B*, giving d*ε*_v_^p^/*d*ε_s_^p^ = *B*/(*A* − *B*/3) when that denominator is nonzero. The parabolic approximation is local to the observed strain interval and must not be extrapolated to arbitrarily large dilation.

### 3.6. Compliance, Coupled Hardening, and Numerical Requirements

For the diagonal prescription (17), the elastoplastic compliance and stiffness are:

(32) $$C^{\mathrm{ep}}\;=\;C^{e}\;+\;K_{1}m_{1}{n_{1}}^{T}\;+\;K_{2}m_{2}{n_{2}}^{T},\;\;D^{\mathrm{ep}}\;=\;{\left(C^{\mathrm{ep}}\right)}^{-1}$$

With associated flow, *m*_i_ = *n*_i_. If C^e^ is positive definite and each active *K*_i_ is non-negative, Equation (32) is symmetric and positive definite. This establishes an invertibility condition for the stated compliance. It does not prove global plastic dissipation, yield-surface convexity, or consistency of the internal-variable update. Those properties require separate examination of the stress domain and hardening law.

In a general two-surface update, the same plastic multiplier may change both shear and volumetric-hardening variables. Write d*α* = *L d*Λ and *H*_ij_ = −(∂*f*_i_/∂α)·L_j_. Active-surface consistency gives *N*^T^d*σ* − *H d*Λ = 0, where *N* = [*n*_1_,*n*_2_]. If *H* is invertible, the general compliance is C^e^ + *P H*^−1^*N*^T^, with *P* = [m_1_,m_2_]. Equation (32) is the special case in which the effective hardening operator is diagonal with entries 1/*K*_i_. The axial and volumetric path-matching construction alone does not establish that diagonality or specify a complete coupled state-update law.

Consequently, a numerical implementation must check the initial hardening state, active-surface selection, derivative accuracy, conditioning of the active system, and non-negativity of admissible multiplier increments. It must update the plastic histories consistently rather than fitting two tangent curves independently at each step. Step-size convergence and unloading–reloading consistency should then be demonstrated at material-point level before finite-element or dam-scale use. No such stress-integration or boundary-value verification is claimed in the present assessment.

### 3.7. Loading and Unloading

Loading cannot be determined by comparing the full yield residual *f*_i_ with its previous maximum, because a consistently active residual remains zero. Let *ψ*_1_ be the stress-dependent part of Equation (5), so that *f*_1_ = *ψ*_1_ − κ_s_; let *ψ*_2_ = *p* + *q*^2^/[*M*_1_^2^(*p* + *p*_r_)], so that *f*_2_ = *ψ*_2_ − *p*_*c*_. Loading history refers to these stress coordinates and their current hardening levels, not to the residuals themselves.

For a state on *f*_i_ = 0, a positive outward stress-induced differential d*σ*f_i_ indicates potential activation, subject to coupled consistency and d*Λ*_i_ ≥ 0. An interior state with *f*_i_ < 0 is elastic with respect to that surface until the surface is reached. Inward motion from a surface gives d*Λ*_i_ = 0 for that unloading increment. When both surfaces are active, both histories must be updated; with one active surface, the inactive multiplier is zero. Complete unloading has *d*Λ_1_ = *d*Λ_2_ = 0 and uses the elastic compliance. This active-set interpretation replaces a maximum-residual criterion and prevents plastic flow during elastic unloading.

### 3.8. Constrained Calibration and Parameter Provenance

Calibration inputs have different evidential status. The axial and volumetric response parameters are fitted to coordinates digitized from the supplied triaxial plots. The unloading ratio *K*_ur_ and logarithmic elastic-compression slope *k* are retained as previously reported parameters and are not newly measured in this study. Contour shape factors are obtained by graphical fitting for the 20 and 100 kg/m^3^ mixtures; the 60 kg/m^3^ shape ratio is a stated interpolation assumption, not an observation. The numerical hardening-knot record required for applying Equation (13) has not been provided. These inputs must not be described collectively as independently verified experimental measurements.

For each binder content, the six axial parameters *E*_0_, *n*, *c*, *φ*, *λ*_0_, and *d*_0_ are fitted jointly across all four pressures using Equation (24). The objective is the sum of the mean squared residual within each pressure series, giving the four pressure series equal weight. Only symbols through the first displayed maximum of each stress series enter this ascending-branch fit. Subsequent symbols remain visible as open markers. The fitted *ε*_m_ must not precede a retained stress point. For the two mixtures with contour records, the fitted strength envelope must also contain those unchanged contour symbols. The resulting *c* and *φ* define effective response targets; without confirmed failure records, they are not independently measured strength constants.

The four volumetric parameters *λ*_1_, *d*_1_, *λ*_2_, and *d*_2_ are calibrated jointly through Equation (28), with *ε*_v_(0) = 0 imposed. All recoverable volumetric symbols in the plotted intervals are included. Positive transition strains, non-negative parameter slopes, and positive maximum-contraction magnitudes are required. These are fitting constraints; a complete image-to-coordinate transformation record and the numerical optimizer bound vectors have not been supplied with this manuscript. Their absence limits independent replication of the digitization and optimization steps. The reported curves describe directly calibrated scalar response functions and are not outputs of a fully integrated two-surface elastoplastic state-update algorithm.

The scalar necessary condition 0 < *E*_t_ ≤ *E* is subsequently checked over the ascending branch. An unloading ratio would require a documented change if that condition were violated; the present constrained response refit satisfies the condition while retaining all three reported *K*_ur_ values. Unloading slopes and radial strains are not recovered by this procedure. The retained *k* and *K*_ur_ values should still be verified against their original records before use in a numerical material model. Table 2 summarizes the parameters used to generate the equations and response curves.

For evaluation, all percentage strains are divided by 100 and quantities reported in 10^−3^ units are multiplied by 0.001. *M*_1_/*M* is a contour-shape factor, additional to the twelve elastic and response parameters. The underlying numerical volumetric-hardening knots have not been included in the recoverable source record, so the piecewise hardening representation is not claimed to be a fully identified constitutive state law.

## 4. Calibration Assessment and Discussion

### 4.1. Experimental Basis and Data Limitations

The calibration data comprise conventional drained triaxial response plots for CSG binder contents of 20, 60, and 100 kg/m^3^ at confining pressures of 300, 600, 900, and 1200 kPa. Digitization provided 378 deviatoric-stress points on the selected ascending intervals and 345 volumetric-strain points; 14 stress points after the first displayed maximum were excluded from axial fitting but retained in the figures. The two available yield-contour panels provided 24 symbols. These graphical points are not new tests, and the twelve binder–pressure conditions do not establish a corresponding number of independent specimens. Specimen identifiers, detailed laboratory logs and independent replicate counts cannot be established from the digitized graphical records. Curve digitization, parameter fitting, and plotting were performed using Origin, version 2024 (OriginLab Corporation, Northampton, MA, USA).

The experimental data considered in this study cover CSG mixtures with binder contents of 20, 60 and 100 kg/m^3^ under confining pressures of 300, 600, 900, and 1200 kPa. The available experimental descriptions report cylindrical specimens approximately 300 mm in diameter and 700 mm in height, prepared using Conch-brand *P*.O. 42.5 ordinary Portland cement (Wuhu Conch Cement Co., Ltd., Wuhu, China) and graded aggregates produced in Liuhe District, Nanjing, China, with a maximum particle size of 40 mm and a water-to-cement ratio of 1.0. The specimens were cured for 28 days, with a temperature of 20 ± 2 °C reported for the strength-test program. Conventional consolidated drained triaxial tests were conducted at an axial displacement rate of 2 mm/min, while separate unloading–reloading tests used a rate of 1 mm/min. The triaxial testing equipment was sourced from the Nanjing Hydraulic Research Institute (Nanjing, China); its specific model designation was not retained in the available records.

### 4.2. Regenerated Response Curves and Calibration Residuals

**Figure 2:**
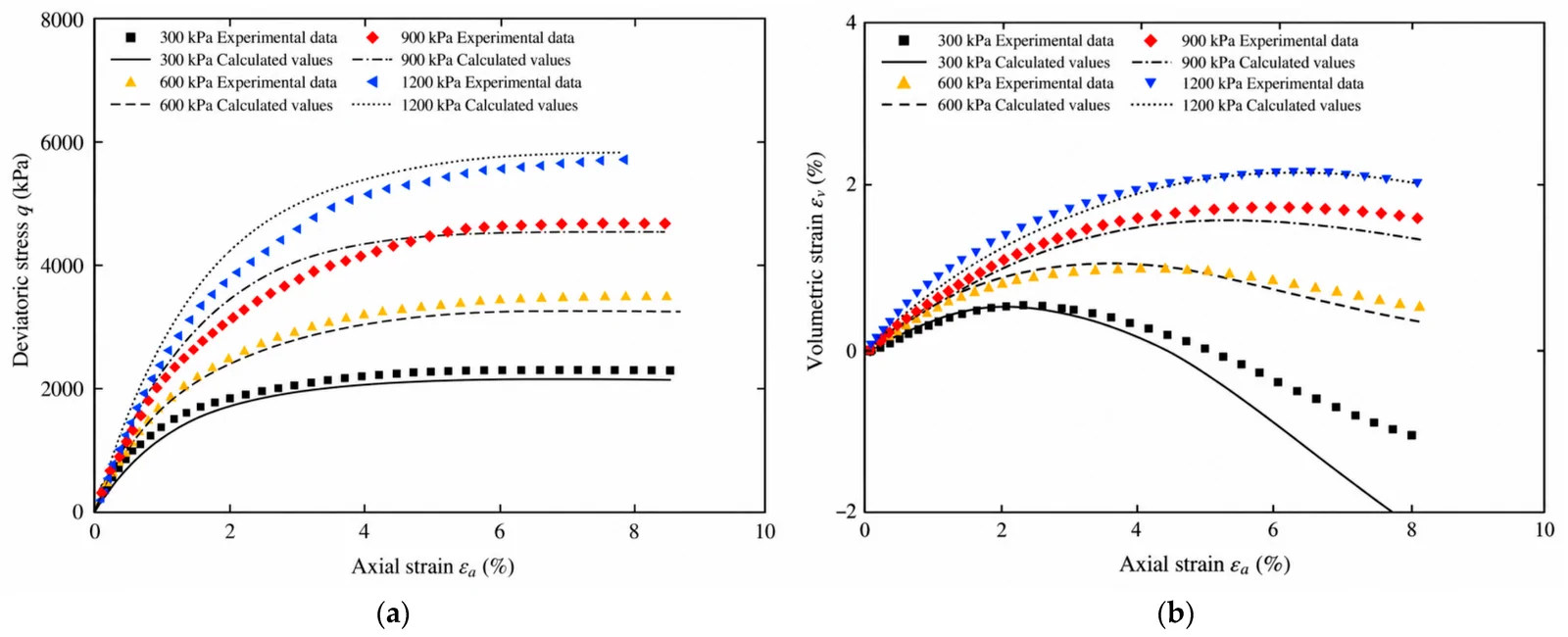
In-sample calibration comparison for CSG with a binder content of 20 kg/m^3^: (**a**) deviatoric stress–axial strain; (**b**) volumetric strain–axial strain. Symbols represent digitized observations, while lines represent fitted response functions at *σ*_3_ = 300, 600, 900 and 1200 kPa. The fitted curves are not independent predictions.

**Figure 3:**
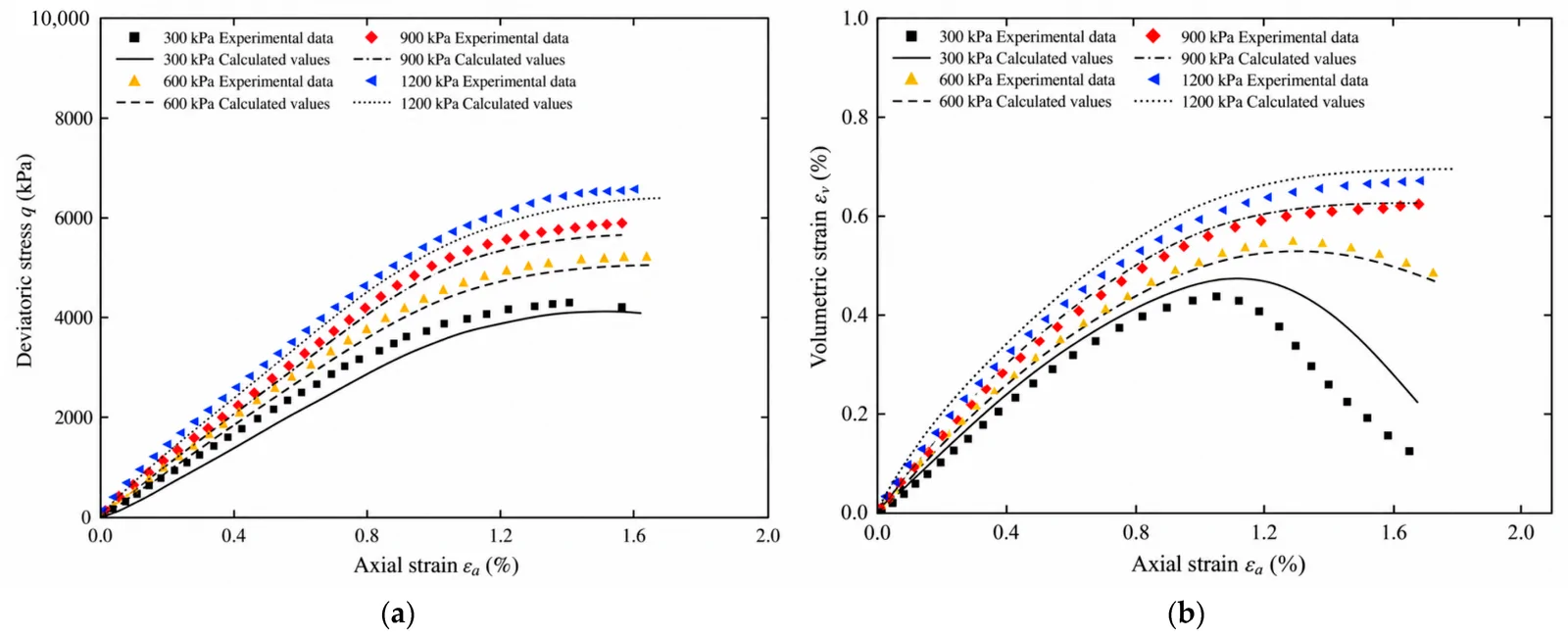
In-sample calibration comparison for CSG with a binder content of 60 kg/m^3^: (**a**) deviatoric stress–axial strain; (**b**) volumetric strain–axial strain. Symbols represent digitized observations, while lines represent fitted response functions at *σ*_3_ = 300, 600, 900, and 1200 kPa. The fitted curves are not independent predictions.

**Figure 4:**
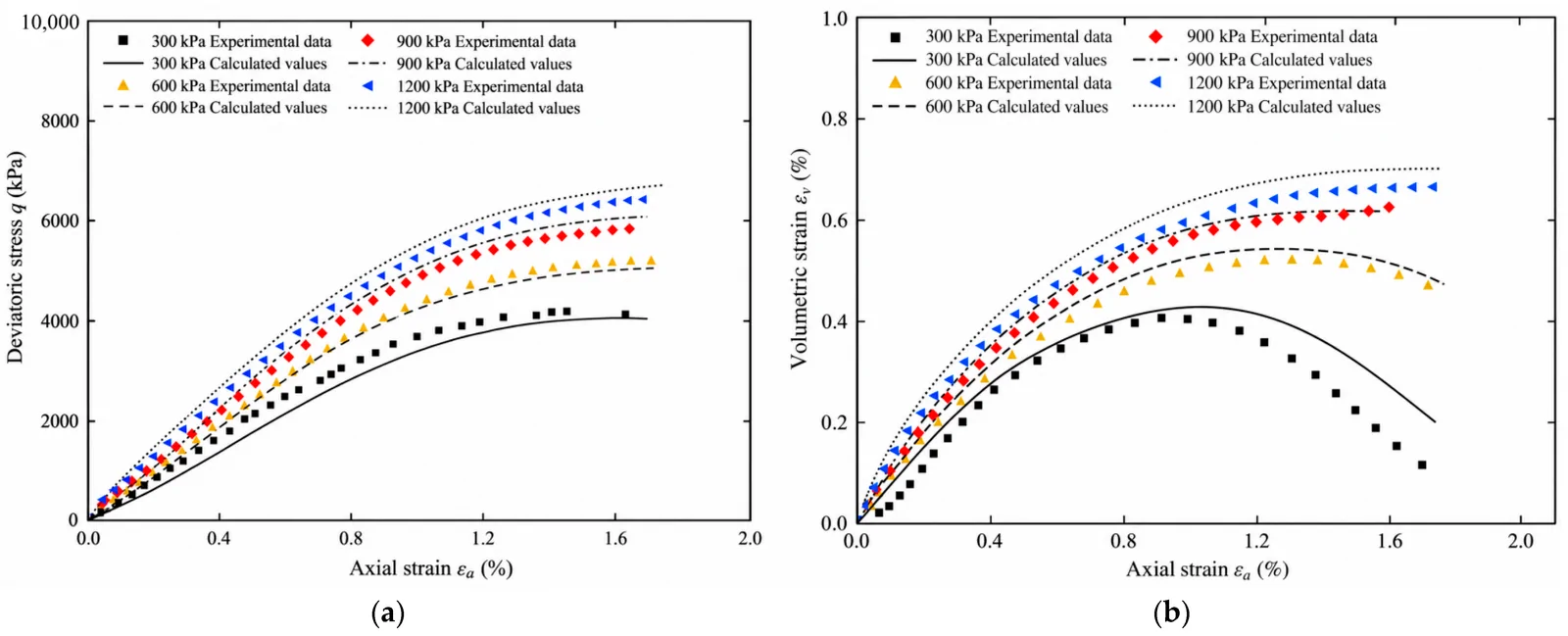
In-sample calibration comparison for CSG with a binder content of 100 kg/m^3^: (**a**) deviatoric stress–axial strain; (**b**) volumetric strain–axial strain. Symbols represent digitized observations, while lines represent fitted response functions at *σ*_3_ = 300, 600, 900 and 1200 kPa. The fitted curves are not independent predictions.

Figure 2, Figure 3 and Figure 4 compare the response-function fits with the available digitized symbols for all four confining pressures. The symbols used for fitting are distinguished from fitted curves; open stress markers denote the 14 points excluded from ascending-branch calibration after the first displayed maximum. Their inclusion for visual context does not establish a measured failure peak or a calibrated post-peak response. The supplied illustrations are raster-based and must be inspected at normal publication size; native-vector artwork and verified source-coordinate files are not available in the current submission. Figure reproduction quality and quantitative fitting accuracy are separate issues.

Table 3 reports pointwise calibration residual statistics. For the *N*q digitized stress observations in a series, the stress root-mean-square error is RMSEq = {Σi[qfit(εa,i) − qdig,i]^2^/*N*q}^1/2^ (kPa), and the mean absolute relative stress error is MAREq = (100/*N*q*)Σi |qfit(εa,i) − qdig,i|/|qdig,i| (%). Fitted values are evaluated at the digitized axial-strain coordinates; the MAREq sum and its count *N*q* include only observations with nonzero |qdig,i|. For the *N*v volumetric observations, RMSEv = {Σi[εv,fit(εa,i) − εv,dig,i]^2^/*N*v}^1/2^ and is expressed in percentage points (pp) when the input strains are percentages. The absolute metric is appropriate when volumetric strain crosses zero. MAE and independently measured peak-stress errors are not reported as numerical results because corresponding pointwise residual lists and confirmed experimental peak values are not supplied for this revision; they cannot be inferred from these RMSE statistics. All reported metrics characterize calibration error only. Different *N*q and *N*v values indicate the number of recoverable graph symbols, not replicate specimen counts.

The stress RMSE values range from approximately 31 to 131 kPa and volumetric RMSE values from 0.021 to 0.179 percentage points across the twelve calibration conditions. The largest volumetric RMSE occurs for the 20 kg/m^3^ mixture at 1200 kPa (0.179 pp), whereas its value at 300 kPa is 0.133 pp. These discrepancies characterize the limited ability of the selected parabolic volumetric response form to match the recovered symbol sequences, but they cannot identify a unique physical cause. For 60 kg/m^3^ at 1200 kPa, the Mohr–Coulomb target used in the fit is not an independently confirmed laboratory peak. Accordingly, neither its signed peak-stress error nor a systematic confinement-dependent peak-strength bias can be established from the available graphical evidence.

The observed stress residuals reflect the fit achieved within the input graphical dataset. They do not demonstrate parameter uniqueness, independent predictive performance, or numerical robustness in a two-surface integration algorithm. In addition, without replicate tests, it is not possible to claim that calibration residuals lie within normal experimental scatter. The *E*_0_–*n* and *c*–*φ* parameter groups may compensate for one another over the limited strain intervals represented by the plots; this issue remains a source of calibration uncertainty.

Related studies have examined CSG dilatancy, binder-dependent deformation, shear strength and unloading–reloading behavior [36,37,38,39]. The volumetric strain–axial strain comparisons in Figure 2b, Figure 3b and Figure 4b describe in-sample fitting behavior. For the low-binder mixture, the digitized response includes comparatively pronounced dilation over part of the displayed strain interval, and the fitted parabola cannot capture every point equally well. At *σ*_3_ = 300 kPa, the volumetric calibration RMSE values are 0.133, 0.032 and 0.029 percentage points for binder contents of 20, 60 and 100 kg/m^3^, respectively (Table 3). They quantify only the discrepancies between graphical symbols and fitted response functions; the absence of repeat tests precludes comparison with true experimental scatter. A possible interpretation of the low-confinement trends is enhanced aggregate rearrangement, including particle sliding, rotation, or overriding. However, this remains a hypothesis rather than a demonstrated micromechanical mechanism because no particle-scale observations are available. The associated flow assumption, imposed parabolic form, potentially different internal states, and unquantified specimen variability are alternative or interacting explanations that cannot be separated using these graphical fits alone.

The formulation prioritizes explicit calibration relations rather than a verified minimum parameter count. Unlike constitutive descriptions with explicit bond damage or hypoplastic degradation [14,15], it does not independently resolve damage to cemented contacts, post-peak softening, cyclic accumulation, rate effects, or aging. Associated flow may influence volumetric fitting and may not represent dilatancy along different loading paths. Although the existing two-surface description allows shear and volumetric terms to be represented separately, its coupled hardening update, numerical implementation, and transferability remain unverified. The results should therefore be limited to the documented ascending response of the investigated conventional drained triaxial paths.

### 4.3. Analytical Parameter Sensitivity

The available parameter relations permit a local analytical sensitivity assessment. This exercise changes one parameter at a time while holding other reported quantities fixed. It evaluates explicit formula dependencies rather than refitting the data or propagating uncertainty through a fully integrated elastoplastic model. The quantities summarized in Table 4 therefore identify immediate parametric effects, without establishing a global sensitivity ranking or model-wide accuracy bounds.

*E*_0_ scales the initial loading modulus directly, while *n* controls its pressure dependence. *K*_ur_ changes the unloading modulus and the available plastic compliance *A*. The elastic bulk modulus is inversely proportional to *k*. Cohesion and friction angle determine the target strength; the friction-angle effect is amplified at higher confinement. The *λ* and *d* pairs control characteristic axial strain, the contraction-dilation transition, and the magnitude of maximum contraction. Their correlation should be retained in uncertainty propagation rather than assuming twelve unrelated errors.

The change in *E*_t_ cannot be inferred from the percentage change in *E*_i_ alone because the shape parameter *β* = *E*_i_ε_m_/*q*_m_ also changes. Similarly, a change in *k* alters elastic strain subtraction and may require refitting the volumetric targets. The listed direct effects should therefore not be combined as if they were independent total-model error bounds.

## 5. Conclusions

A double-yield-surface incremental description for CSG has been recalibrated and assessed under the reported conventional drained triaxial conditions. The following findings are supported by the available evidence.

(1) The shear-volumetric separation and the inherited shear construction are distinguished from the binder-dependent response extension. Stress-induced yield differentials, full consistency differentials, plastic multipliers, and path-matching coefficients are now defined separately. The rational interpolation coefficients and triaxial reduction have explicit derivations.

(2) In-sample graphical fits are assessed for 378 deviatoric-stress points on selected ascending intervals and 345 volumetric-strain points. Their stress RMSE values are 31–131 kPa, and their volumetric RMSE values are 0.021–0.179 percentage points. These statistics represent digitized-point calibration errors, not independent validation, specimen-to-specimen variability, or numerical peak-stress prediction accuracy.

(3) The constrained recalibration resolves the identified axial inconsistency of the 60 kg/m^3^ set. Its maximum *E*_t_/*E* falls from values above one to 0.913, 0.854, 0.833, and 0.824 at the four pressures, with the reported unloading ratio retained. All recalibrated sets pass this necessary axial check and have admissible inferred elastic constants. Coupled coefficient positivity and numerical state-update consistency remain additional requirements.

(4) The available 20 and 100 kg/m^3^ contour plots support mixture-specific graphical shape estimates, while the 60 kg/m^3^ shape ratio is an explicitly assumed interpolation. Because numerical volumetric-hardening knots and original experimental provenance cannot be established from the supplied record, neither a fully transferable hardening law nor an independently verified particle-scale mechanism is demonstrated. Further source-data recovery, repeated tests, and independent stress-path experiments are needed before the formulation can be used as a verified engineering material model.

## Figures and Tables

**Table 1:** Constitutive features of relevant CSG models and their relation to the present formulation.

Model	Surfaces, Mechanisms, and Binder Treatment	Calibration and Comparison Scope
Cai et al. [11] (2016)	Shear and volumetric yield surfaces; equal-plastic-strain construction; nine reported parameters.	Reported assessment for 60 kg/m^3^ CSG. The double-surface foundation and shear construction are inherited here.
Fu et al. [14] (2022)	Damage hypoplasticity with cementation degradation; not the same yield-surface construction.	Requires material and damage characterization. Explicit bond damage is absent from the present hardening description.
Ren et al. [15] (2023)	Strength-parameter evolution and statistical damage; addresses cementation-related degradation.	Damage and strength data underpin calibration. A common-dataset accuracy comparison is not available.
Wu et al. [21] (2025)	Strength-based CSG model; shear and volumetric surfaces; softening and dilation included.	Double yielding is shared. The present study does not demonstrate greater post-peak or dilatancy capability.
Present formulation	Associated potentials; binder-specific axial and volumetric tangent fits; 12 response and elastic parameters, fitted volumetric shape factors, and a volumetric-hardening interpolation framework without recovered numerical knots.	Drained shear, axial unloading–reloading, and isotropic unloading–reloading characterization; three binder contents, σ_3_ = 300–1200 kPa.

**Table 2:** Parameters used to generate the revised equations and curves.

Parameter	Reporting Unit	20 kg/m^3^	60 kg/m^3^	100 kg/m^3^
*E*_0_	dimensionless	1554.74	2072.37	3158.76
*n*	dimensionless	0.267475	0.399409	0.362277
*φ*	degrees	41.3829	34.1493	34.0447
*c*	kPa	246.788	946.906	997.689
*K*_ur_	dimensionless	2	1.3	1.08
*λ*_0_	%	0.0589329	0.0238895	0.0321244
*d*_0_	%	7.85741	1.49992	1.81313
*λ*_1_	10^−3^	4.42304	0.650128	0.546451
*d*_1_	%	1.5506	0.645651	0.700675
*λ*_2_	10^−3^	1.90871	0.34904	0.321695
*d*_2_	%	0.001	0.321363	0.316702
*k*	%	0.26	0.21	0.12
*M*_1_/*M*	dimensionless	1.3469	1.25867 (assumed)	1.17044

**Table 3:** Pointwise calibration residuals against digitized graphical observations; RMSE denotes root-mean-square error, MARE denotes mean absolute relative error, and pp denotes percentage points.

Binder kg/m^3^	*σ*_3_ kPa	*N*q	*N*v	Stress RMSE kPa	Stress MARE %	Volume RMSE pp
20	300	29	34	31	1.6	0.133
20	600	34	30	34	1.5	0.127
20	900	37	36	55	1.7	0.083
20	1200	38	36	67	3.5	0.179
60	300	28	26	40	2.6	0.032
60	600	28	25	48	2.9	0.037
60	900	35	23	84	3.3	0.026
60	1200	42	25	84	3.8	0.055
100	300	24	28	71	2.9	0.029
100	600	25	23	131	6.1	0.035
100	900	26	28	80	3.9	0.021
100	1200	32	31	89	3.4	0.042

**Table 4:** Local sensitivities obtained analytically from the explicit parameter relations (not a full-model uncertainty analysis).

Quantity	Parameter Change or Derivative	Direct Effect
*E*_i_	*E*_0_ +10%, with *n* fixed	*E*_i_ +10% at every confinement.
*E*_i_	*n* +0.05	*E*_i_ +7.18% at 300 kPa; +13.68% at 1200 kPa.
*E*_i_	*n* −0.05	*E*_i_ −6.70% at 300 kPa; −12.04% at 1200 kPa.
*E* = *K*_ur_*E*_i_	*K*_ur_ +10%, *E*_i_ fixed	*E* +10%; *E*_t_ is unchanged in the fixed-target response interpolation; *A* changes.
*K* = (*p*_0_ + *P*_a_)/*k*	*k* +10%/*k* −10%	*K* −9.09%/*K* +11.11% at fixed *p*_0_.
*q*_m_	∂*q*_m_/∂*c* = 2cos φ/(1 − sin φ)	Strength increases linearly with *c* at fixed *φ* and *σ*_3_.
*ε*_m_; *ε*_n_; *ε*_*vd*_	∂*ε*/∂*λ* = *σ*_3_/*P*_a_; ∂*ε*/∂*d* = 1	Slope uncertainty is amplified by confinement; intercept uncertainty shifts the response target.
Volumetric surface term	*M*_1_ +10%/*M*_1_ −10%	*q*^2^/[*M*_1_^2^(*p* + *p*_r_)] changes by −17.36%/+23.46%, with *p* and *q* fixed.
Volumetric–hardening relation *H*_v_	No numerical parameter perturbation is reported	A sensitivity curve cannot be evaluated without verified pressure–plastic-strain knots and the initial hardening state.

## Data Availability

The original contributions presented in this study are included in the article. Further inquiries can be directed to the corresponding author.

## Appendix A. Algebraic Relations Underlying the Triaxial Calibration

### Appendix A.1. Isotropic Elastic Identities

For linear isotropic elasticity, *E* = 3*K*(1 − 2*ν*). Rearrangement gives *ν* = 1/2 − *E*/(6*K*), as used in Equation (3). The relation follows from the isotropic elastic moduli and does not represent an independent fitted law.

### Appendix A.2. Fixed-Confinement Strength Target

On a constant-*σ*_3_ triaxial compression path, *p* = *σ*_3_ + *q*/3. Combining this with *q* = *M*(*p* + *p*_r_), *M* = 6 sin φ/(3 − sin φ), and *p*_r_ = *c* cot φ gives *q* [1 − *M*/3] = *M*(*σ*_3_ + *p*_r_). Hence *q*_m_ = 2(*c* cos φ + *σ*_3_ sin φ)/(1 − sin φ). This is the theoretical path-envelope intersection, not automatically an observed laboratory peak.

### Appendix A.3. Rational Axial-Response Coefficients

Let *q*(*ε*_a_) = *ε*_a_/(*a*_h_ + *b*_h_ε_a_ + *c*_h_ε_a_^2^) as in Equation (23). The initial condition d*q*/d*ε*_a_ at *ε*_a_ = 0 equals *E*_i_, so *a*_h_ = 1/*E*_i_. Setting the derivative to zero at *ε*_a_ = *ε*_m_ gives *c*_h_ = *a*_h_/*ε*_m_^2^. Finally, *q*(*ε*_m_) = *q*_m_ gives *b*_h_ = 1/*q*_m_ − 2/(*E*_i_ε_m_). These expressions reproduce Equation (25) for the prescribed ascending-branch target.

### Appendix A.4. Reduction in the Two Active-Surface Coefficients

For fixed radial stress, d*p* = d*q*/3 and d*σ*_1_ = d*q*. Using the active-surface prescription in Equation (17), the axial and volumetric incremental compliances become d*ε*_a_/d*q* = 1/*E* + *K*_1_*R*_1_^2^ + *K*_2_*R*_2_^2^ and d*ε*_v_/d*q* = 1/(3*K*) + *K*_1_*R*_1_*R*_3_ + *K*_2_*R*_2_*R*_4_. Equating these with 1/*E*_t_ and μ_t_/*E*_t_ yields the linear system in Equation (21). Solving it gives the coefficients in Equation (22), provided *R*_1_*R*_2_(*R*_1_*R*_4_ − *R*_2_*R*_3_) ≠ 0. This algebra alone does not establish admissible plastic multipliers or a stable coupled state update.
